# Mechanistic Insights into Nitroarene Hydrogenation Dynamics on Pt(111) via *In Situ* Tip-Enhanced Raman Spectroscopy

**DOI:** 10.1021/jacs.5c14338

**Published:** 2025-10-17

**Authors:** Zhen-Feng Cai, Meghna A. Manae, Zi-Xi Tang, Anastasiia Moskalenko, Yao Zhang, Jeremy O. Richardson, Naresh Kumar

**Affiliations:** † Key Laboratory of Green Chemistry and Technology of Ministry of Education, College of Chemistry, 12530 Sichuan University, 29 Wangjiang Road, Chengdu 610064, China; ‡ Department of Chemistry and Applied Biosciences, 27219 ETH Zurich, Vladimir-Prelog-Weg 3, Zurich CH-8093, Switzerland; § Hefei National Laboratory for Physical Sciences at the Microscale and Synergetic Innovation Center of Quantum Information and Quantum Physics, 12652 University of Science and Technology of China, Hefei 230026, Anhui, China

## Abstract

Mechanistic insights into the molecular-level dynamics of nitroarene hydrogenation on Pt remain limited, largely because most prior studies rely on *ex situ*, ensemble-averaged measurements, or simulations considered in isolation. Here, we address this gap and demonstrate a novel methodology combining *in situ* tip-enhanced Raman spectroscopy (TERS) with density functional theory (DFT) modeling to track, at a well-defined single plasmonic junction, the hydrogenation of chloronitrothiophenol (CNTP) on atomically flat Pt(111). *In situ* TERS captures the dynamic transformation of CNTP → chloroaminothiophenol (CATP) under ambient H_2_ exposure with a characteristic time scale of ∼6 s. Complementary DFT modeling maps the reaction energetics, revealing novel mechanistic insights: CNTP desorption is rapid initially (barrier 0.61 eV) but slows down once the Pt(111) surface is at about half-coverage; molecular bending on the half-covered Pt(111) surface is barrierless and exergonic; the first hydrogen addition to CNTP is facile (barrier 0.26 eV), while the second hydrogen addition is kinetically most demanding (barrier 0.83 eV), yielding a time scale of seconds that matches experimental results and identifies the rate-determining step. These findings advance molecular-level understanding of nitroarene hydrogenation on Pt(111) and demonstrate *in situ* TERS integrated with first-principles DFT modeling as a powerful platform for operando mechanistic studies of heterogeneous catalytic processes at the nanoscale.

## Graphical abstract

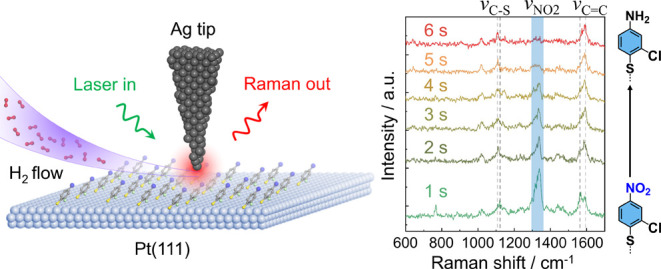

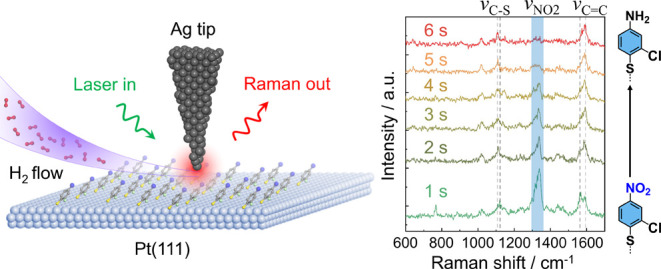

## Introduction

The catalytic hydrogenation of nitroarenes to anilines is a fundamental transformation in synthetic chemistry, underpinning the manufacture of pharmaceuticals, dyes, agrochemicals, and polymer precursors.^1–4^ Despite its apparently simple stoichiometry, the reaction proceeds through a complex network of elementary steps and short-lived intermediates such as nitroso and hydroxylamine species,^5–10^ making the identification of the operative pathway and the rate-determining step challenging. Multiple mechanisms have therefore been proposed for hydrogenation of substituted nitroarenes under different conditions.^5–8,11,12^ However, much of the available insight derives either from *ex situ*, ensemble-averaged measurements performed on large populations of variably sized catalytic nanoparticles, which obscure site-specific dynamics, or from theoretical calculations considered in isolation, lacking direct experimental validation. Consequently, the molecular-level dynamics governing nitroarene hydrogenation under working conditions remain largely unresolved.

Direct, real-time observation of catalytic transformations under working conditions is essential for establishing rigorous mechanistic understanding.^13–17^ Operando spectroscopies including X-ray photoelectron,^18–21^ infrared,^22–24^ nuclear magnetic resonance,^25–27^ and Raman^28–30^ allow probing of catalytic processes with chemical specificity, providing ensemble-level insights. However, these methods often lack the sensitivity or spatial resolution required to resolve site-specific activity and dynamics at the nanometer scale. Tip-enhanced Raman spectroscopy (TERS) addresses this gap by combining single-molecule sensitivity and chemical specificity of surface-enhanced Raman scattering with the spatial precision of scanning probe microscopy, enabling chemically selective readout with nanometer resolution under ambient conditions.^31–34^ TERS provides direct nanoscale vibrational fingerprints of reactants, intermediates, and products in well-defined molecular systems, typically probing fewer than 100 molecules in the "gap-mode" plasmonic near-field,^35^ and has been successfully applied to study a range of catalytic transformations.^28,30,36–41^ However, to the best of our knowledge, hydrogenation of nitroarene on Pt catalysts has not been tracked *in situ* using TERS yet.

In contrast to previous studies, herein we employ a highly controlled model system, a well-defined, close-packed chloronitrothiophenol (CNTP) monolayer on an atomically flat Pt(111) surface and monitor its hydrogenation dynamics at a single plasmonic junction using *in situ* TERS. We demonstrate that this approach yields highly reproducible data and, when combined with complementary density functional theory (DFT) modeling, provides more definitive mechanistic insights. *In situ* TERS reproducibly captures both the onset and temporal evolution of the transformation of CNTP to chloroaminothiophenol (CATP) under ambient H_2_ exposure, yielding a characteristic hydrogenation time scale of approximately 6 s. Complementary periodic DFT calculations map the energetics along the reaction coordinate, allowing direct comparison of calculated energy barriers with the measured reaction rate. The experimental and computational results establish a consistent mechanistic picture in which CNTP desorption competes with hydrogenation on Pt(111) surface; the first hydrogen addition within the nitro group is facile, while the second hydrogen addition is kinetically most demanding and constitutes the rate-determining step, in agreement with the observed reaction time scale. Beyond advancing the mechanistic understanding of nitroarene hydrogenation on Pt surfaces, this work demonstrates that *in situ* TERS integrated with first-principles DFT modeling is a powerful platform for operando, nanoscale mechanistic studies of heterogeneous catalytic reactions.

## Results and Discussion

Figure 1 illustrates the experimental setup employed for *in situ* monitoring of CNTP hydrogenation on the Pt(111) surface. Scanning tunneling microscopy (STM) imaging confirmed the atomically flat morphology of the Pt(111) single crystal (Figure S1). Prior to H_2_ exposure, hyperspectral TERS imaging was carried out to assess the uniformity and extent of CNTP adsorption on the Pt(111) surface. 100 spectra measured in a TERS map acquired over a 1 μm × 1 μm area are shown as a waterfall plot in Figure 2a. The spectra exhibit characteristic vibrational signatures of CNTP molecules.^42^ The relatively uniform TERS intensity observed across the mapped region indicates a homogeneous surface coverage of CNTP on the Pt(111) surface. Notably, no far-field Raman signal was detected from the CNTP/Pt(111) sample under 632.8 nm laser excitation in the absence of the Ag tip (Figure S2), highlighting the monolayer sensitivity of TERS measurements.

**Figure 1:**
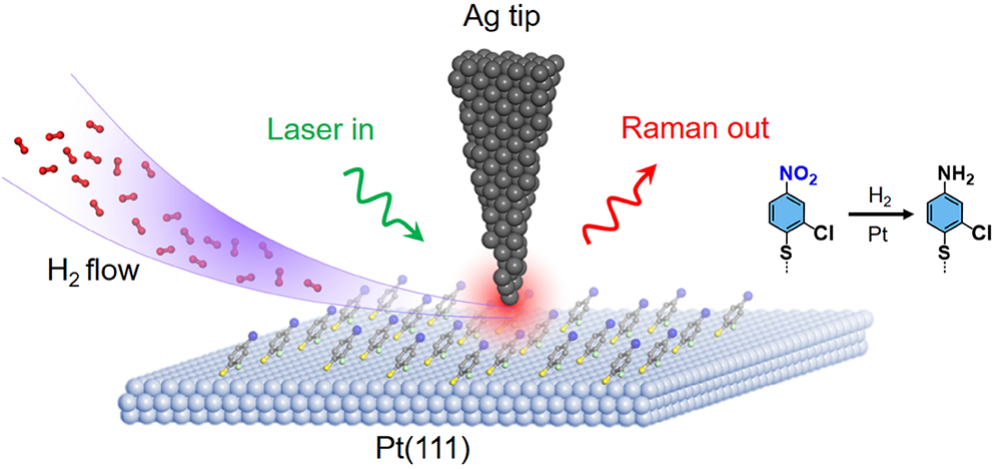
Schematic of the STM–TERS platform for *in situ* monitoring of nitroarene hydrogenationon Pt(111). A reflection-mode STM-TERS configuration is used to follow the hydrogenation of CNTP to CATP on Pt(111) under ambient conditions. The CNTP-functionalized Pt(111) surface is maintained in tunneling contact with a Ag tip, forming a gap-mode plasmonic junction while a continuous H_2_ flow is supplied. Localized surface plasmon resonance (LSPR) at the Ag tip apex generates a highly intense and localized electromagnetic field in the junction, enabling nanoscale *in situ* TERS readout of the reaction at a single hotspot.

**Figure 2:**
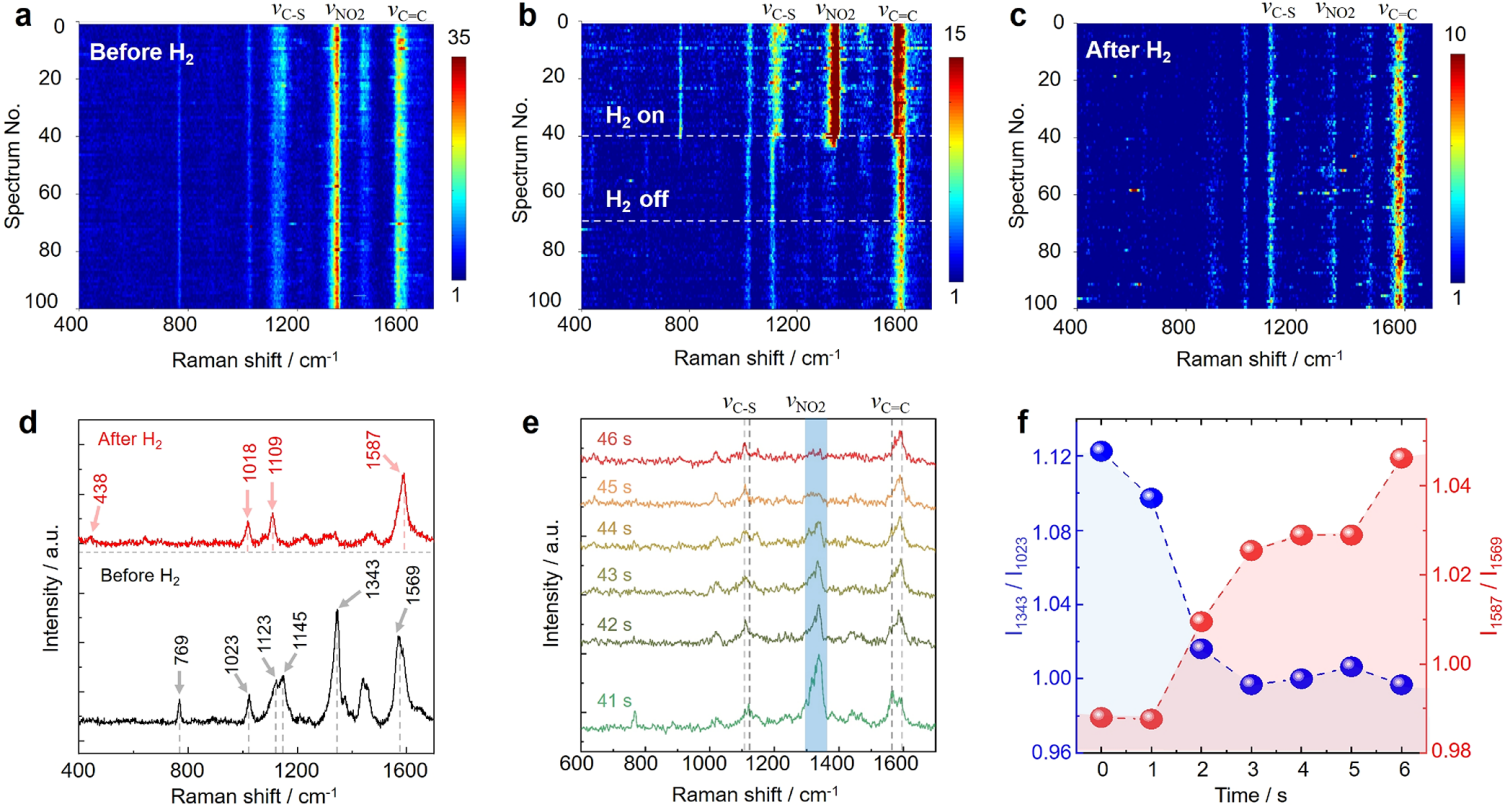
*In situ* monitoring of catalytic hydrogenation of CNTP on Pt(111) surface. (a) Waterfall plot of 100 TERS spectra acquired across a 1 × 1 μm^2^ area of the CNTP/Pt(111) sample before H_2_ exposure. Step size: 100 nm. Spectrum integration time: 1 s. (b) Waterfall plot of 100 time-sequenced TERS spectra recorded at a fixed location on the CNTP/Pt(111) sample before, during, and after H_2_ exposure. Hydrogen gas was introduced over the sample between 40 and 70 s (total duration: 30 s), as indicated in the plot. Spectrum integration time: 1 s. (c) Waterfall plot of 100 TERS spectra acquired across a 1 × 1 μm^2^ area of the CNTP/Pt(111) sample after H_2_ exposure. Step size: 100 nm. Spectrum integration time: 1 s. (d) Averaged TERS spectra of CNTP/Pt(111) sample before and after H_2_ treatment from panel (b) exhibiting characteristic Raman modes of CNTP and CATP, respectively. (e) TERS spectra showing real-time molecular transformation of CNTP during catalytic hydrogenation on Pt(111). (f) Temporal evolution of intensity ratios *I*_1343_/*I*_1023_ and *I*_1587_/*I*_1569_, corresponding to the NO_2_ and C=C vibrational modes, respectively.

We next applied *in situ* STM-TERS to monitor the catalytic hydrogenation of CNTP on Pt(111) under the reaction conditions. Figure 2b displays waterfall plots of 100 sequential TERS spectra acquired at a fixed location on the CNTP/Pt(111) surface, collected before, during, and after H_2_ exposure with an integration time of 1 s per spectrum. H_2_ with a low flow rate of ∼0.1 L/s was introduced over the CNTP/Pt(111) sample for a duration of 30 s, spanning from 40 to 70 s, as indicated in Figure 2b. Immediately upon introduction of H_2_ into the tip–sample gap, a rapid decrease in the intensity of the NO_2_ symmetric stretching mode at 1343 cm^–1^ was observed, accompanied by spectral shifts at 1023, 1123, and 1569 cm^–1^ as depicted in the waterfall plot. These spectral changes indicate the onset of the catalytic hydrogenation of surface-anchored CNTP molecules (Figure 2c). Averaged TERS spectra acquired before and after H_2_ exposure (Figure 2d) and assignment of the observed Raman modes based on literature (Table S1) confirm the CNTP → CATP conversion, exhibiting characteristic vibrational markers of nitro-group hydrogenation.^42^

To visualize the temporal evolution of the hydrogenation process, six TERS spectra acquired between 41 and 46 s (immediately following H_2_ introduction) are shown in Figure 2e. Before H_2_ exposure, TERS signals observed at 1127, 1343, and 1567 cm^–1^ correspond to the C–S stretching, NO_2_ symmetric stretching, and C=C stretching modes of CNTP, respectively.^42^ Upon introduction of H_2_, a rapid decrease in the intensity of the NO_2_ mode at 1343 cm^–1^ is observed, along with a red shift of the C–S mode (from 1123 to 1109 cm^–1^) and a blue shift of the C=C mode (from 1569 to 1587 cm^–1^). These spectral changes reflect the chemical transformation of CNTP to CATP^42^ and are quantitatively captured in the time-dependent intensity ratios *I*_1343_/*I*_1023_ and *I*_1587_/*I*_1569_ displayed in Figure 2f, which show decreasing and increasing trends, respectively. Notably, the most significant spectral transitions occur within the first 5 s after H_2_ exposure, with the NO_2_ mode completely disappearing and the C–S and C=C bands shifting to their final positions by the sixth second. Notably, upon H_2_ exposure, an overall attenuation of the Raman signals is also observed. This signal decrease is attributed to hydrogen-induced cleavage of Pt–S bonds, indicating that dissociated H atoms on the Pt(111) surface not only promote nitro group hydrogenation but also facilitate desorption of CNTP molecules by weakening the thiol–metal interaction. The observed thiol-desorption is consistent with our previous findings^43^ and also aligns with observations reported by Yin et al.^42^ under similar experimental conditions.

In long-duration TERS measurements, sample drift is a commonly encountered challenge.^44^ To enable reliable nanoscale spectroscopy, drift-compensation strategies have been established.^45,46^ For our STM-TERS setup, the measured drift rate is rather low (0.02–0.03 nm s^–1^).^47^ Given this low drift rate, together with the low H_2_ flow rate, drift-induced artifacts are expected to be negligible over the short time scale of the time-dependent TERS measurements in this study.

To verify the reproducibility of the observed hydrogenation dynamics, *in situ* TERS measurements were repeated on an independent CNTP/Pt(111) sample subjected to 28 s of H_2_ exposure. The corresponding data and analysis are presented in Figure S3. In agreement with the results shown in Figure 2e, complete conversion of CNTP to CATP was observed within 7 s following H_2_ introduction, confirming the reproducibility of the reaction time scale under comparable experimental conditions.

To determine whether the observed hydrogenation of CNTP is catalyzed by Pt(111) or driven by laser illumination or plasmon-induced hot electrons in the TERS near-field, we performed control *in situ* TERS experiments on a CNTP-functionalized Au(111) surface under identical conditions. As shown in Figures S4–S6, the TERS spectra acquired before, during, and after H_2_ exposure exhibit no discernible changes in vibrational features, indicating the absence of any hydrogenation reaction on Au(111). These results confirm that neither laser illumination nor plasmonically generated hot electrons in the TERS near-field is sufficient to drive the reaction. CNTP-to-CATP hydrogenation requires surface hydrogen generated by H_2_ dissociation on Pt(111)^48^ (whereas H_2_ does not spontaneously dissociate on Au), highlighting the essential catalytic role of Pt(111) in this transformation.

An intriguing feature of the CNTP/Au(111) system is the absence of azo bond formation between adjacent nitro groups during TERS measurements, a process commonly observed for 4-nitrothiophenol on Au surfaces.^36,50,51^ The TERS spectra collected before, during, and after H_2_ exposure display a high signal-to-noise ratio (Figure S5), confirming a strong plasmonic enhancement at the Ag tip apex under 632.8 nm excitation. This rules out insufficient plasmonic enhancement as the cause of suppressed dimerization. Instead, steric hindrance from the bulky Cl substituent likely prevents interactions between adjacent nitro groups, thereby inhibiting dimercaptoazobenzene (DMAB) formation, in agreement with our previous findings.^37^

A key question is whether hydrogenation proceeds exclusively at the NO_2_ group of CNTP during the *in situ* reaction or whether the C–Cl bond is also susceptible to reduction under our experimental conditions. In other words, which is the dominant hydrogenation product −CATP or dechlorinated 4-aminothiophenol (4ATP)? To address this, we performed DFT calculations of the Raman spectra of CNTP, CATP, and 4ATP molecules adsorbed on a Pt(111) nanocluster. The optimized molecular structures and their corresponding simulated spectra are presented in Figure 3. For comparison, we also included the experimental TERS spectra of the CNTP/Pt(111) sample recorded before (red) and after (blue) *in situ* H_2_ exposure. The DFT-calculated vibrational features of CNTP on Pt(111) closely match the TERS spectrum of the reactant.

**Figure 3:**
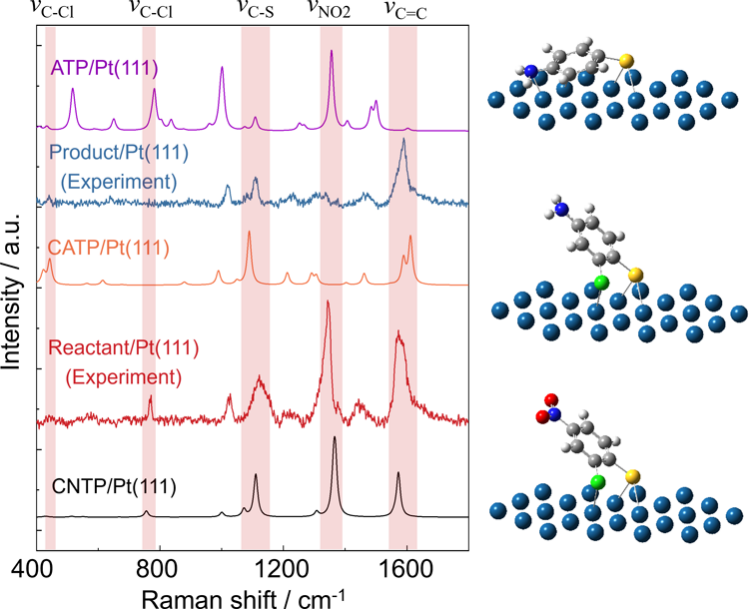
Comparison of the experimental and DFT-calculated Raman spectra of CNTP hydrogenation on Pt(111). Averaged TERS spectra acquired from the CNTP/Pt(111) sample before (red) and after (blue) *in situ* exposure to H_2_. DFT-calculated Raman spectra for CNTP (black), CATP (orange), and 4ATP (purple) adsorbed on the (111) surface of a Pt nanocluster for vibrational mode assignment and identification of the hydrogenation product. *Inset:* Optimized molecular structures of CNTP, CATP, and 4ATP adsorbed on the (111) surface of the Pt nanocluster used in the DFT calculations. Atom color scheme: Pt (cyan), C (gray), H (white), S (yellow), Cl (green), N (blue), O (red). Importantly, the experimental spectrum of the product shows significantly better agreement with the calculated Raman spectrum of CATP/Pt(111) than that of 4ATP/Pt(111), confirming CATP as the primary hydrogenation product. In particular, the presence of the C–Cl stretching mode is identified in both reactant and product spectra. In the experimental and DFT-simulated spectra, this mode exhibits a rather large shift from 755 cm^–1^ in CNTP to 421 cm^–1^ in CATP, which indicates that the C–Cl bond remains intact following hydrogenation, ruling out dechlorination of the molecule. The pronounced red shift is likely attributable to the high sensitivity of the C–Cl vibrational mode to molecular structure, adsorption configuration, and local electronic interactions, which are markedly altered during hydrogenation.^52,53^

To quantify the extent of hydrogenation, we performed a comparative analysis of averaged experimental TERS spectra collected before and after H_2_ exposure and the DFT-calculated spectra of CNTP and CATP adsorbed on Pt(111). The detailed analysis procedure is provided in the Experimental Section of the Supporting Information. This analysis reveals that upon H_2_ treatment, roughly half of the CNTP molecules desorb from the Pt(111) surface, while the remaining fraction undergoes hydrogenation to form CATP. The partial desorption suggests that the adlayer reorganizes into a lower-coverage thermodynamically more stable self-assembled monolayer. This stabilization at reduced packing density is supported by calculated desorption energies, which show stronger binding at 44% coverage (2.73 eV, 3 × 3 cell) compared to 100% coverage (0.61 eV, 2 × 2 cell), in agreement with the experimental observations.

Dynamical TERS measurements revealed that the transformation from CNTP to CATP under ambient conditions occurs within approximately 6 s (Figures 2e and S3b). To gain mechanistic insight into the rate-determining step, we complemented these experimental findings with periodic DFT calculations of the hydrogenation pathway. Geometry optimization of CNTP on a 2 × 2 Pt(111) unit cell showed that the molecule adsorbs in a tilted geometry, with an inclination of ∼65° relative to the surface. For effective hydrogen abstraction from the Pt surface, the NO_2_ group must approach closer, requiring significant bending of the CNTP molecule. However, this conformational flexibility is sterically hindered in the densely packed pristine CNTP self-assembled monolayer, as illustrated in Figure S7. Partial desorption of adjacent molecules reduces steric constraints, enabling the necessary bending and facilitating reaction progression. These insights prompted us to investigate whether the desorption and associated molecular bending steps represent kinetic bottlenecks in the overall hydrogenation mechanism.

To assess whether CNTP desorption constitutes the rate-determining step, we performed periodic DFT modeling of the desorption process with a CNTP molecule on a 2 × 2 Pt(111) surface unit cell. For the CNTP/Pt(111) sample, this coverage represents a self-assembled monolayer with a nearly 100% packing density. In our previous study, we demonstrated that while H_2_ spontaneously undergoes dissociative adsorption on the Pt(111) surface,^48^ plasmonically generated hot electrons within the TERS near-field can further enhance H_2_ dissociation by up to 20%, resulting in a nonequilibrium overpopulation of hydrogen atoms on the Pt(111) surface.^43^ On the Pt(111) surface, adsorption of dissociated hydrogen atoms at bridge sites is energetically more favorable than at top sites by 0.44 eV.^43^ However, under the high local flux of H_2_ and the plasmon-enhanced H_2_ dissociation in the TERS near-field, not only the bridge sites but also a substantial fraction of the top sites are expected to become populated. Despite their lower stability, hydrogen atoms at the top sites exhibit markedly higher reactivity due to the reduced energy barriers. For instance, the desorption time scale of a thiol molecule (PhSH) from Pt(111) via reaction with an adsorbed H atom was found to be approximately 8 h at bridge sites versus only 6 ms at top sites.^43^ Given the experimentally observed hydrogenation time scale of ∼6 s for CNTP → CATP, we infer that both desorption and hydrogenation proceed predominantly via reaction with top-site H adatoms on Pt(111). Accordingly, periodic DFT simulations of the desorption and hydrogenation pathways were performed with H adsorbed at the top sites. As the calculated energy profile in Figure S8 shows, the energy difference between the adsorbed and gas-phase desorbed states of CNTP is 0.61 eV with no barrier exceeding this value along the minimum-energy path, indicating that adsorption is energetically favored (also see Supporting GIF 1). However, desorption is entropically driven and nonreversible. The desorption rate is estimated by applying the Arrhenius equation:

1 $$k=A\;e^{-E_{a}/k_{B}T}$$

Assuming a typical pre-exponential factor of *A* = 10^13^ s^–1^ for the desorption process^54^ and a reaction temperature of 300 K, the rate constant (*k*) is estimated to be approximately 600 s^–1^. This corresponds to a characteristic time scale (1/*k*) of ∼2 ms, indicating that desorption occurs far too rapidly to serve as the rate-determining step for hydrogenation of CNTP to CATP under ambient conditions.

We next investigated the bending of CNTP molecules toward the Pt surface, a structural rearrangement essential for enabling interaction between the NO_2_ group and surface-bound hydrogen atoms. In the densely packed 2 × 2 self-assembled monolayer model, this bending is geometrically constrained by steric hindrance from neighboring molecules (Figure S7). However, given that desorption removes approximately half of the adsorbed CNTP species, we modeled the bending and subsequent reaction steps on a more dilute 3 × 3 Pt(111) surface unit cell, as shown in Figure S9 (also see Supporting GIF 2). Upon structural relaxation, the bent CNTP configuration was found to be energetically favored by 0.74 eV compared to the nearly upright geometry, indicating a thermodynamically more stable adsorption state. Climbing-image nudged elastic band (CI-NEB) calculations show that the bending transition proceeds without an energy barrier, confirming that this conformational change does not represent the rate-determining step. Notably, on the more crowded 2 × 2 surface, this bent geometry could not be stabilized, suggesting that partial desorption is a prerequisite for the remaining CNTP molecules to adopt the reactive, bent conformation necessary for efficient hydrogenation.

To elucidate the hydrogenation mechanism, we computed the reaction energy profile for the stepwise conversion of CNTP to CATP on Pt(111) using periodic DFT, fully optimizing the key intermediates and transition states identified in prior nitroarene hydrogenation studies.^6,7,11,12^ The resulting energy profile is shown in Figure 4a. A schematic representation of the proposed hydrogenation pathway, highlighting the key elementary steps and intermediates involved, is presented in Figure 4b. The overall stoichiometry of the reaction can be represented as

2 $$\mathrm{CNTP}+\mathrm{6H}\to \mathrm{CATP}+2H_{2}O$$

**Figure 4:**
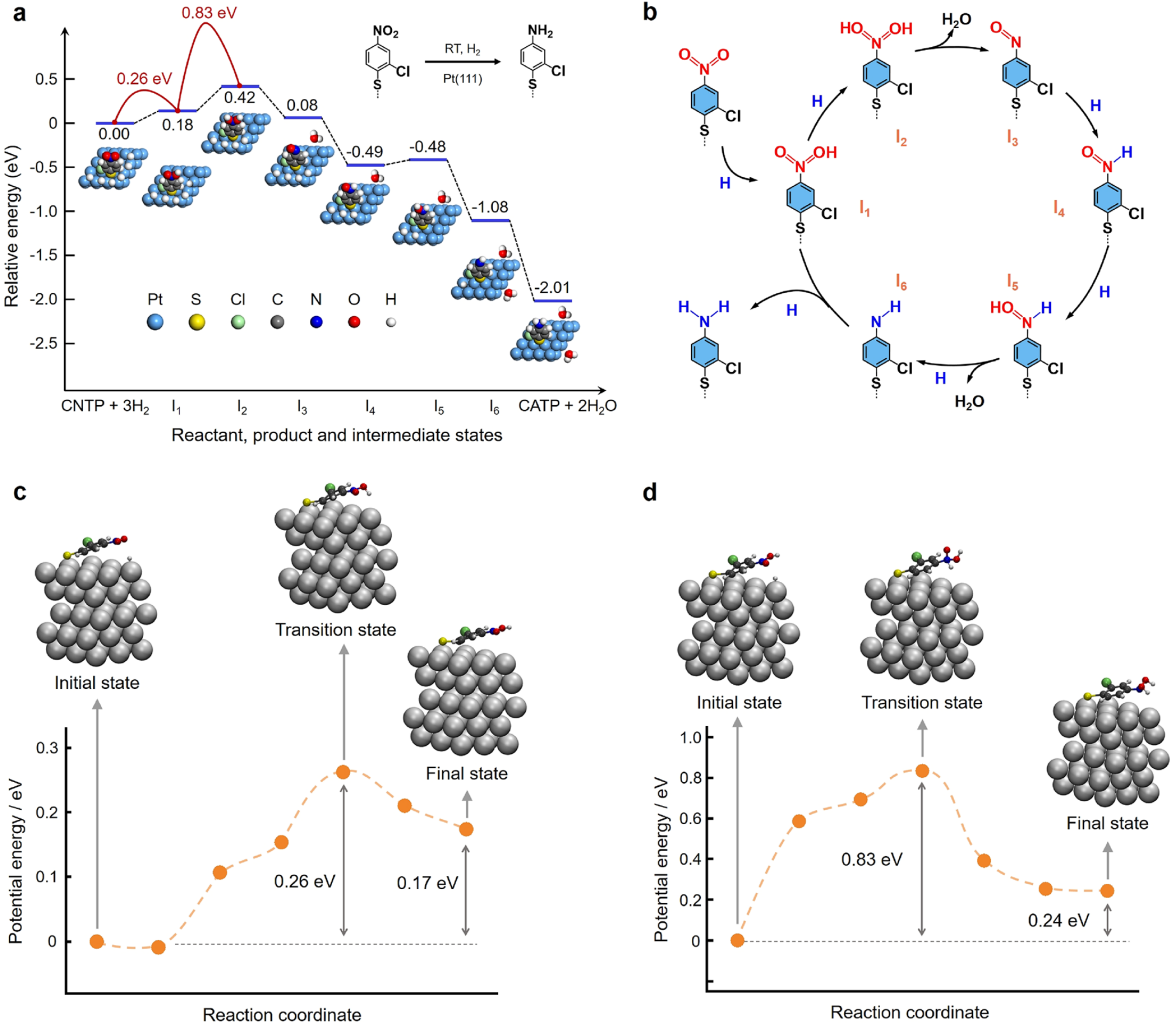
Reaction energy profile for the catalytic hydrogenation of CNTP to CATP on Pt(111). (a) Periodic DFT-calculated energy landscape for the stepwise hydrogenation of CNTP to CATP on the Pt(111) surface. The relative energies of the key intermediates (*I*_1_–*I*_6_) and the final product CATP/Pt(111), are referenced to the initial CNTP/Pt(111) state. The activation energy barriers are indicated in red. All energies are reported in eV. Schematic representations of the hydrogenation steps are shown beneath the calculated energy profile. Atom color scheme in panel (a): Pt (cyan), C (gray), S (yellow), Cl (green), N (blue), O (red), H (white). (b) Schematic illustration of the proposed hydrogenation pathway of CNTP on Pt(111) shown in panel (a), highlighting the key elementary steps and intermediates involved in the catalytic transformation. (c) Calculated energy profile for the abstraction of the first H atom by a CNTP molecule from a top site on the Pt(111) surface, modeled using a 3 × 3 unit cell. The initial, intermediate, and final states derived from the CI-NEB calculations are also shown. (d) Calculated energy profile for the abstraction of a second H atom by a CNTP molecule from a top site on the Pt(111) surface, modeled using a 3 × 3 unit cell. The initial, intermediate, and final states derived from the CI-NEB calculations are also shown. Atom color scheme in panel (c, d): Pt, gray; C, black; H, white; S, yellow; Cl, green; N, blue; O, red.

The initial state consists of one CNTP molecule and six hydrogen atoms adsorbed on the Pt(111) surface. It is important to note that this configuration is not energetically equivalent to a system comprising CNTP and three gas-phase H_2_ molecules, as it includes the additional stabilization energy associated with hydrogen adsorption on the metal surface. To quantify this adsorption energy, we calculated the stabilization per hydrogen atom using the following expression:

$$\Delta E_{H}=E_{\mathrm{CNTP}+H}-\left(E_{\mathrm{CNTP}}+\frac{1}{2}E_{H_{2}}\right)$$ 3

where *E*_CNTP+H_ is the energy of CNTP coadsorbed with a single hydrogen atom on Pt(111), *E*_CNTP_ is the energy of CNTP adsorbed alone, and *E*_H_2__ is the energy of an isolated gas-phase hydrogen molecule. This yielded a stabilization energy of −0.454 eV per hydrogen atom. To confirm the consistency of this value, we performed an analogous calculation using the first hydrogenated intermediate (*I*_1_ in Figure 4a,b), as follows

$$\Delta E_{H}=E_{I_{1}+H}-\left(E_{I_{1}}+\frac{1}{2}E_{H_{2}}\right)$$ 4

where *E*_I__1__+H_ is the energy of first intermediate structure *I*_1_ and 1H atom adsorbed on Pt(111), and *E*_I_1__ is the energy of *I*_1_ adsorbed on Pt(111). This yielded a comparable stabilization energy of −0.475 eV. Taking the average of the two results (Δ*E*_H_ = −0.465 eV), we defined the effective energy of an adsorbed hydrogen atom as

$$E_{H}=\Delta E_{H}+\frac{1}{2}E_{H_{2}}$$ 5

Assuming that H_2_O desorbs immediately upon formation, its energy was taken directly from the gas-phase calculation.

These reference energies were used to construct the reaction energy profile for the transformation of CNTP to CATP on Pt(111) (Figures 4a and S10). The energy landscape reveals that the initial two steps, corresponding to the formation of intermediates *I*_1_ and *I*_2_, are endotherm *ic*, while the subsequent transformations are predominantly exothermic except for the *I*_4_ to *I*_5_ step being mildly endothermic. The pronounced stabilization of intermediates *I*_3_ and *I*_6_ is attributable to the exergonic formation of H_2_O. While the energy profile provides thermodynamic insight, a complete mechanistic understanding also requires an evaluation of the kinetic barriers associated with the elementary steps.

Since the first two hydrogenation steps are endothermic, we explicitly calculated activation energies for these steps leading to the formation of intermediates *I*_1_ and *I*_2_, which are shown in Figure 4c,d, respectively. Subsequent steps are expected to be rapid, consistent with their predominantly exothermic character and correspondingly lower activation energies. For formation of *I*_1_, the barrier is 0.26 eV, which, using a standard pre-exponential factor,^54^ corresponds at 300 K to a reaction rate of 4.0 × 10^8^ s^–1^ and a characteristic time scale of ∼2.5 ns (Figure 4c; Supporting GIF 3). This is several orders of magnitude faster than the experimentally observed hydrogenation time scale of ∼6 s, ruling out this step as rate-limiting. In contrast, formation of *I*_2_ proceeds over a 0.83 eV barrier, giving a rate constant of 0.098 s^–1^ and a characteristic time scale of ∼10 s (Figure 4c; Supporting GIF 4), in reasonable agreement with our experimental results (Figures 2 and S3). These results identify the second hydrogenation step (*I*_1_ → *I*_2_) as the rate-determining step in the overall catalytic sequence.

## Conclusions

In summary, using STM-based *in situ* TERS, we tracked the CNTP → CATP hydrogenation in real time at a single plasmonic hotspot on a well-defined Pt(111) surface. TERS spectra captured spectral evolution of key vibrational modes during hydrogenation and revealed a characteristic reaction time scale of ∼6 s. CATP was confirmed as the dominant reaction product through literature assignment as well as first-principles DFT calculation of the Raman vibrational modes. Furthermore, periodic DFT-calculated energy landscape for the stepwise hydrogenation of CNTP to CATP on the Pt(111) surface provided further mechanistic insights. Desorption of CNTP from Pt(111) was found to occur rapidly, whereas bending of CNTP toward the Pt(111) surface was found to be barrierless. Importantly, the time scale of the addition of second hydrogen to the nitro group was found to be ∼10 s, matching the experimental result and identifying it as the rate-determining step. Although the current temporal resolution of ∼1 s is sufficient to reliably capture hydrogenation dynamics occurring on the time scale of seconds, we envisage that future advances in tip fabrication that provide higher enhancement factors could enable shorter integration times. This would allow the detection of short-lived intermediates and the investigation of faster reaction dynamics without compromising spectral quality. The findings of this study advance molecular-level understanding of nitroarene hydrogenation on Pt(111). Additionally, this work demonstrates that *in situ* TERS, integrated with first-principles DFT modeling, can deliver site-specific, dynamic mechanistic insights on a nonplasmonic catalyst. By bridging the gap between nanospectroscopy, surface science, and theoretical modeling, this approach could open new avenues for a deeper understanding of heterogeneous catalytic processes.

## Supplementary Material





## Data Availability

The original data used in this publication are made available in a curated data archive at ETH Zurich (https://www.researchcollection.ethz.ch) under the DOI: 10.3929/ethz-c-000782927. Correspondence and requests for materials should be addressed to Z.F.C., J.O.R., or N.K.

## Notes

Experimental section; STM images of Pt(111) and Au(111); far-field Raman spectrum of CNTP/Pt(111); assignments of Raman modes; additional dataset demonstrating in situ monitoring of CNTP hydrogenation on Pt(111); *in situ* TERS results of CNTP/Au(111) samples; and periodic DFT results of CNTP desorption and bending on Pt(111) (PDF)
CNTP desorption pathway reconstructed from the nine CI-NEB images; CNTP bending pathway reconstructed from the CI-NEB image; first H-abstraction pathway reconstructed from the CI-NEB images; and second H-abstraction pathway reconstructed from the CI-NEB images (ZIP)
