# Supplementary material for: Mechanistic Insights into Nitroarene Hydrogenation Dynamics on Pt(111) via In Situ Tip-Enhanced Raman Spectroscopy
Source: J Am Chem Soc. 2025 Oct 17;147(43):39838–45. doi: 10.1021/jacs.5c14338 (PMC12576771; doi:10.1021/jacs.5c14338)
Supplement: Supplementary file 1 [file ja5c14338_si_001.pdf]

Supplementary Information

Mechanistic Insights into Nitroarene Hydrogenation Dynamics on Pt(111) via In Situ Tip-Enhanced Raman Spectroscopy

Zhen-Feng Cai[1,2]*, Meghna A. Manae[2], Zi-Xi Tang[3], Anastasiia Moskalenko[2], Yao Zhang[3], Jeremy O. Richardson[2]*, and Naresh Kumar[2]*

[1]Key Laboratory of Green Chemistry and Technology of Ministry of Education, College of Chemistry, Sichuan University 29 Wangjiang Road, Chengdu 610064, China
[2]Department of Chemistry and Applied Biosciences, ETH Zurich, Vladimir-Prelog-Weg 3, CH-8093 Zurich, Switzerland.
[3]Hefei National Laboratory for Physical Sciences at the Microscale and Synergetic Innovation Center of Quantum Information and Quantum Physics, University of Science and Technology of China, Hefei, Anhui 230026, China.

*Email: caizf@scu.edu.cn (Z.F.C.), jeremy.richardson@phys.chem.ethz.ch (J.O.R.), naresh.kumar@org.chem.ethz (N.K.)

Materials and Methods

Sample preparation

Chloronitrothiophenol (CNTP) solution was prepared by dissolving the solid sample in ethanol and further dilution to generate concentration series. The Pt(111) single-crystal substrates were prepared by the Clavilier method.[1] To prepare CNTP/Pt(111) sample, freshly prepared Pt(111) substrates were immersed in a 1 mM solution of CNTP in ethanol for 1 hour and then rinsed thoroughly using ethanol.

TERS setup and measurements

TERS measurements were conducted using a top-illumination configuration that integrates a scanning tunneling microscope (STM) with a Raman spectrometer (NT-MDT, Russia). A 100× air objective with a numerical aperture of 0.7 (Mitutoyo, Japan) facilitated both the excitation and collection of TERS signals. Electrochemically etched silver tips were employed for STM and TERS experiments.[2] The excitation source was a 632.8 nm He-Ne laser (Newport, Germany) delivering 70 μW of power at the sample surface. During all STM-TERS measurements, the tunneling current and bias voltage were maintained at 200 pA and 0.5 V, respectively. For *in situ* H_2 treatment, a copper pipe was positioned approximately 5 mm from the tip-sample gap. To enable precise control during hydrogenation reactions on the surface, a flow meter was installed outside the STM-TERS system's environmental control chamber. During H_2 treatment, the hydrogen flow rate was adjusted to 10 L/min.

DFT calculations of Raman spectra

The calculations of optimized molecular structures and vibrational properties of the CNTP, chloroaminothiophenol (CATP), and 4-aminothiophenol (ATP) adsorbed on (111) surface of a Pt nanocluster of 27 atoms were performed with the Gaussian 16 program package[3] using the hybrid PBE0 functional with a mixed basis set, i.e., the C, H, N, S, O and Cl atoms with the 6-311++G (d, p) basis set[4] and Pt atoms with the SDD basis set.[5] The substrate contains three layers with 28 Pt atoms and the scale factor of vibrational frequency is 0.9612.

Periodic DFT calculations of CNTP to CATP conversion on Pt(111)

Periodic DFT calculations of the reaction pathways were performed using the Quantum ESPRESSO[6] code with the optB88-vdW functional.[7] This functional has been known to accurately account for van der Waals interactions[8] and has been previously used to study a system similar to the one in this work.[9] Kinetic energy cut-offs of 45 Ry and 550 Ry were imposed on the wavefunctions and charge density, respectively. Structures were determined through minimization of energy until the Hellmann–Feynman forces on each atom were smaller than 0.03 eV/Å. The Pt (111) slab was modelled with 5 layers with a vacuum of at least 15 Å above the surface and the bottom three planes were frozen in all optimizations. The desorption reaction was modelled with a 2×2 unit cell and a k-point mesh of 6×6×1. All other reactions and intermediates were

modelled with a 3×3 unit cell and a k-point mesh of 3×3×1. The climbing image nudged elastic band (CI-NEB) method was used to obtain reaction barriers with 7 images to model the reactions with a force optimization threshold of 0.05 eV/Å. Cartesian coordinates and cell parameters for all the optimized structures and CI-NEB paths are provided in the Supplementary Information.

Estimation of CNTP desorption from Pt(111) after H_2 treatment

The extent of CNTP desorption from the Pt(111) surface following H_2 exposure was estimated through a comparative analysis of experimental TERS spectra and DFT-calculated Raman spectra of CNTP and CATP adsorbed on Pt(111). The procedure was as follows: (i) Averaged TERS spectra were collected from the CNTP/Pt(111) sample before and after H_2 exposure. (ii) The ν(C–S) and ν(C=C) marker bands were identified, and their peak areas were integrated after baseline correction. (iii) The integrated peak areas were normalized to their respective DFT-derived Raman cross-sections to account for differences in intrinsic scattering efficiencies. (iv) The reduction in normalized peak areas of the ν(C–S) and ν(C=C) bands after H_2 exposure was converted into desorption percentages. (v) The desorption values obtained from the two marker bands were averaged to determine the final desorption ratio. The ν(C–S) and ν(C=C) modes were selected as marker bands because of their well-defined intensities and minimal spectral overlap with other vibrational features.

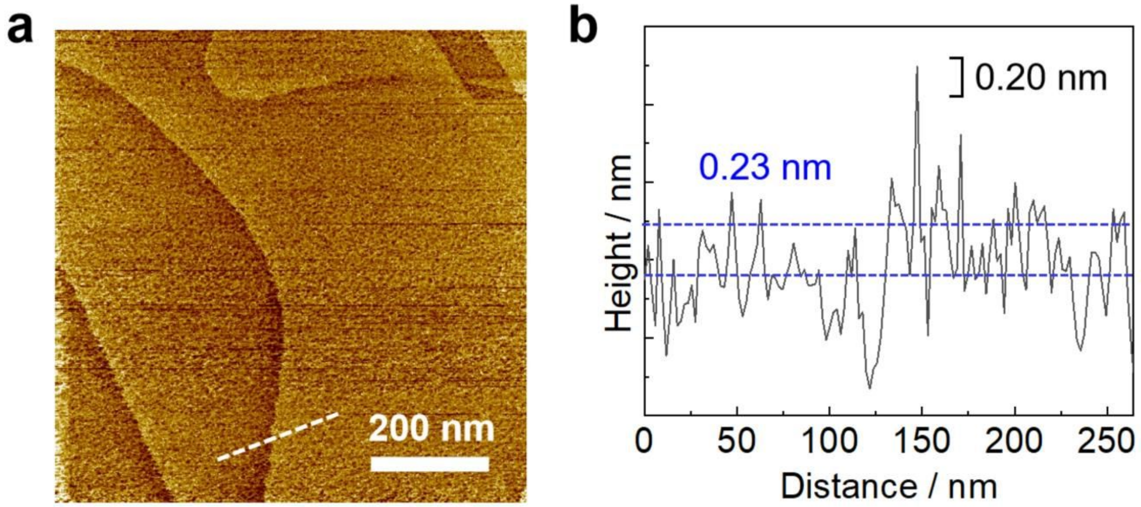

Figure S1. Topographical characterization of the Pt(111) surface. (a) Representative STM topography image of a pristine Pt(111) substrate used in this work. (b) The cross-section profile shows topography variation across a step-edge along the line marked in Panel a, confirming the atomically flat nature of the Pt(111) surface.

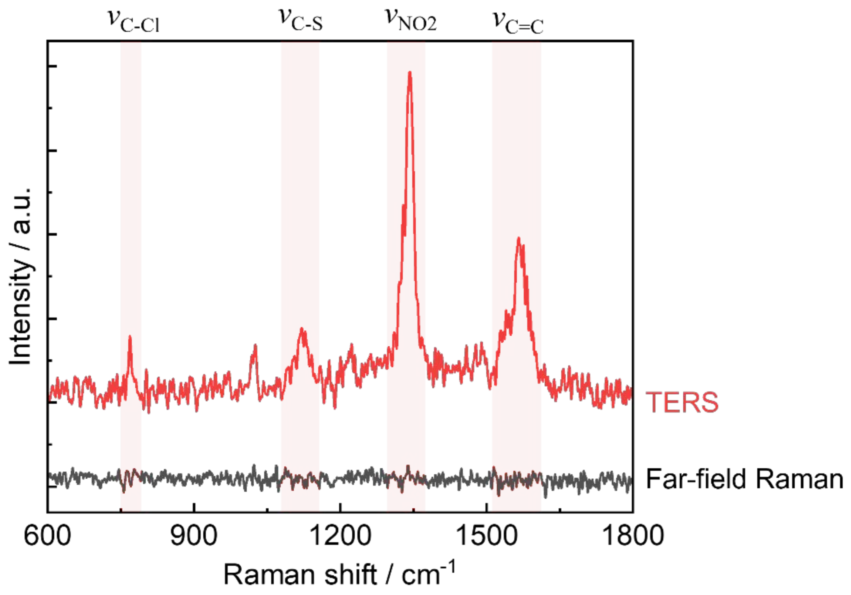

Figure S2. Comparison of TERS and far-field Raman spectra of CNTP/Pt(111) sample. TERS (red) and far-field Raman (black) spectra of a CNTP/Pt(111) sample acquired under the same experimental conditions. Distinct vibrational bands characteristic of CNTP are readily observed in the TERS spectrum, whereas no detectable features are present in the far-field Raman spectrum. This contrast underscores the monolayer-level molecular sensitivity of TERS under ambient conditions.

Table S1. Tentative vibrational assignments of Raman modes for the reactant (CNTP) and hydrogenation product (CATP) observed in the averaged TERS spectra recorded before and after H_2 exposure (Figure 2d).

Reactant (CNTP)				Product (CATP)			
Raman shift / cm^{-1}	Calculated / cm^{-1}	Literature[9] / cm^{-1}	Assignment	Raman shift / cm^{-1}	Calculated / cm^{-1}	Literature[9] / cm^{-1}	Assignment
769	756	765	$\nu_{(C\text{-}Cl)}$	438	441	–	$\nu_{(C\text{-}Cl)}$
1023	1000	1024	α_{ring}	1018	990	1024	α_{ring}
1123	1067	1027	$\nu_{(C\text{-}S)}$	1109	1090	1108	$\nu_{(C\text{-}S)}$
1145	1109	–	$\nu_{(C\text{-}N)}$	–	–	–	–
1343	1365	1336	$\nu_{(NO2)}$	–	–	–	–
1569	1571	1561	$\nu_{(C=C)}$	1587	1610	1586	$\nu_{(C=C)}$

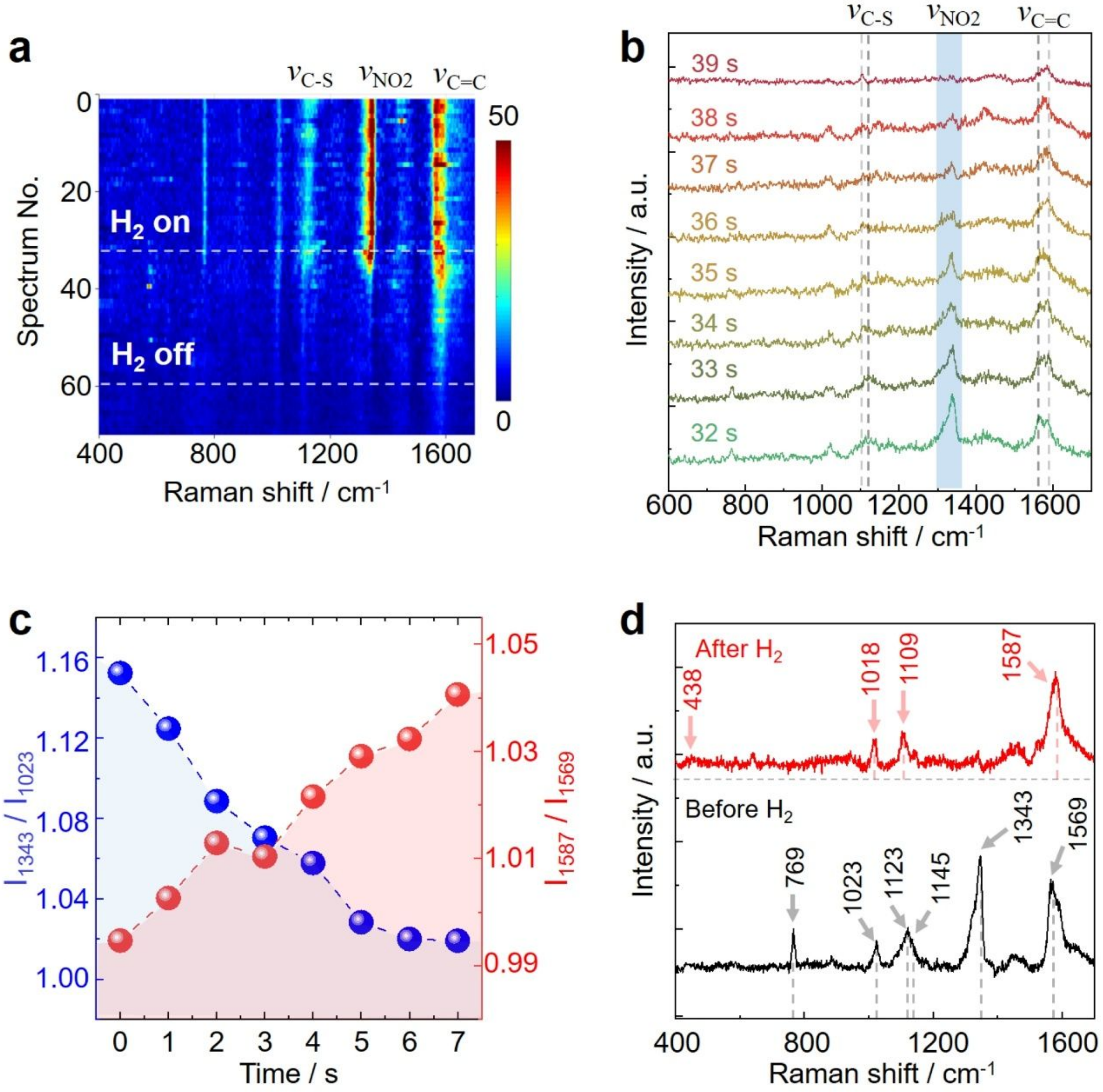

Figure S3. Additional dataset demonstrating in situ monitoring of CNTP hydrogenation on Pt(111). (a) Waterfall plot of 70 time-sequenced TERS spectra acquired at a fixed location on the CNTP/Pt(111) sample before, during, and after H_2 exposure. Hydrogen gas was introduced into the tip–sample gap between 32 and 60 seconds, corresponding to a total exposure duration of 28 seconds. Each spectrum was recorded with an integration time of 1 s. (b) Dynamical TERS spectra highlighting the catalytic hydrogenation of CNTP molecules on Pt(111) under reaction conditions. The transformation is completed within 7 seconds, similar to the timescale observed in Figure 2d, which confirms the reproducibility of the observed hydrogenation dynamics. (c) Temporal evolution of the intensity ratios I_{1343}/I_{1023} and I_{1587}/I_{1569}, corresponding to the NO_2 and C=C vibrational modes, respectively. (d) Averaged TERS spectra of CNTP/Pt(111) sample before and after H_2 treatment exhibiting characteristic Raman modes of CNTP and CATP, respectively.

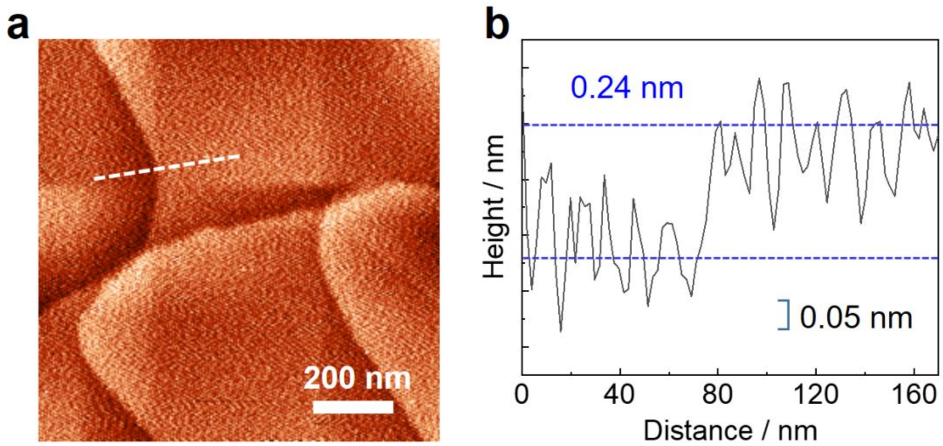

Figure S4. Topographical characterization of the Au(111) substrate. (a) Representative STM topography image of a pristine Au(111) substrate used in this study. (b) Cross-sectional height profile across the step edge along the line marked in Panel a, confirming the atomically flat nature of the Au(111) surface.

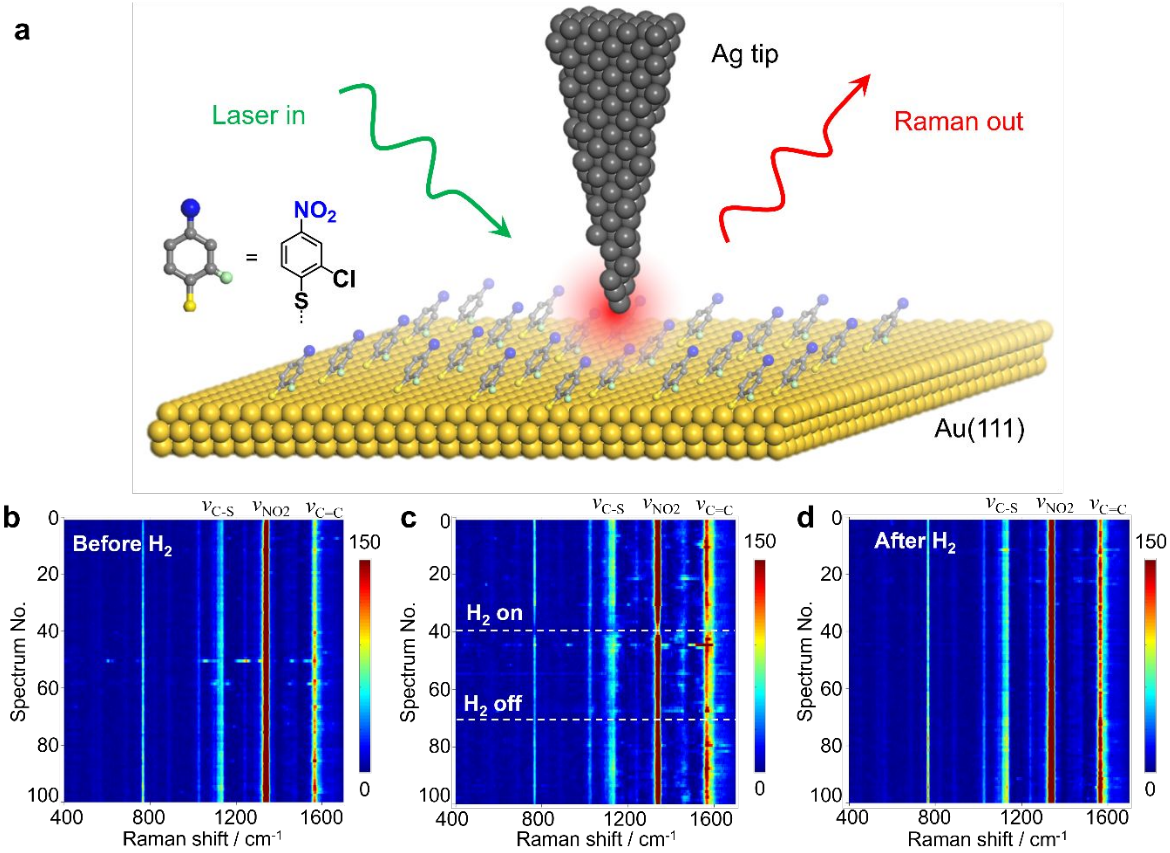

Figure S5. Control in situ TERS measurements of a CNTP/Au(111) sample before, during, and after H_2 exposure. (a) Schematic illustration of the STM-TERS setup used to probe the CNTP-functionalized Au(111) surface under in situ conditions. The STM image of the Au(111) surface shown in Figure S4 confirms its atomically flat topography. (b) Waterfall plot of 100 TERS spectra acquired in a 1 × 1 μm^2 TERS map of the CNTP/Au(111) sample prior to H_2 introduction. Step size: 100 nm. Spectrum integration time: 1 second. (c) Waterfall plot of 100 sequential TERS spectra recorded at a fixed location on CNTP/Au(111) sample before, during, and after H_2 exposure. H_2 gas was introduced over the CNTP/Au(111) sample from 41 to 70 seconds, for a total duration of 30 seconds. Spectrum integration time: 1 second. (d) Waterfall plot of 100 TERS spectra acquired in a 1 × 1 μm^2 TERS map of the CNTP/Au(111) sample after H_2 exposure. Step size: 100 nm. Spectrum integration time: 1 second.

Averaged TERS spectra of the CNTP/Au(111) sample before, during, and after H_2 exposure are presented in Figure S6, which show no detectable spectral changes before, during, or after H_2 exposure, indicating that, under identical experimental conditions, CNTP does not undergo hydrogenation on Au(111) surface. These control experiments confirm neither laser illumination nor plasmon-induced hot electrons in the TERS near-field are sufficient to drive this reaction in the absence of dissociated hydrogen atoms, which validates the essential catalytic role of Pt in facilitating the hydrogenation of CNTP to CATP.

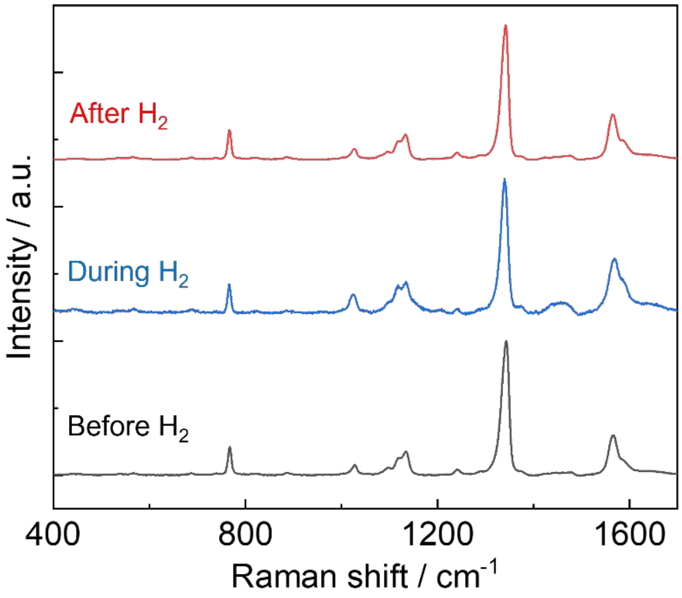

Figure S6. Averaged TERS spectra of the CNTP/Au(111) sample before, during, and after H_2 exposure. Averaged TERS spectra corresponding to the in situ TERS measurements presented in Figure S5c were extracted to evaluate potential spectral changes before, during and after H_2 treatment of the CNTP/Au(111) surface. All three spectra are virtually identical, exhibiting absence of any detectable vibrational changes.

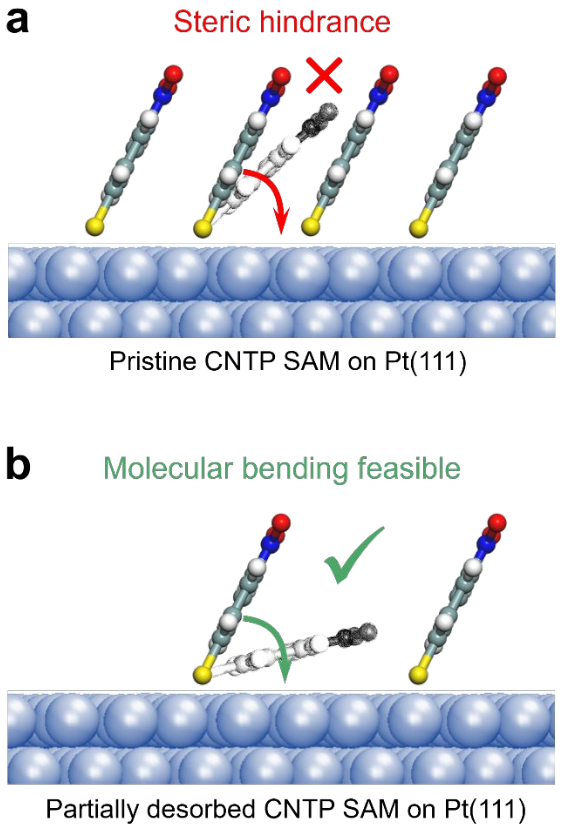

Figure S7. Schematic diagram illustrating the steric hindrance effect influencing CNTP hydrogenation on Pt(111). (a) Schematic representation of the pristine CNTP self-assembled monolayer on Pt(111), where steric hindrance from adjacent CNTP molecules restricts the molecular bending required for effective interaction between the $–NO_2$ group and surface-bound hydrogen atoms. (b) Upon partial desorption of CNTP molecules, reduced intermolecular crowding allows the remaining CNTP molecules to adopt a bent conformation, thereby facilitating interaction between the $–NO_2$ group and hydrogen atoms on the Pt(111) surface necessary for hydrogenation. Atom color scheme: light blue, Pt; yellow, S; white, H; green, C; blue, N; red, O.

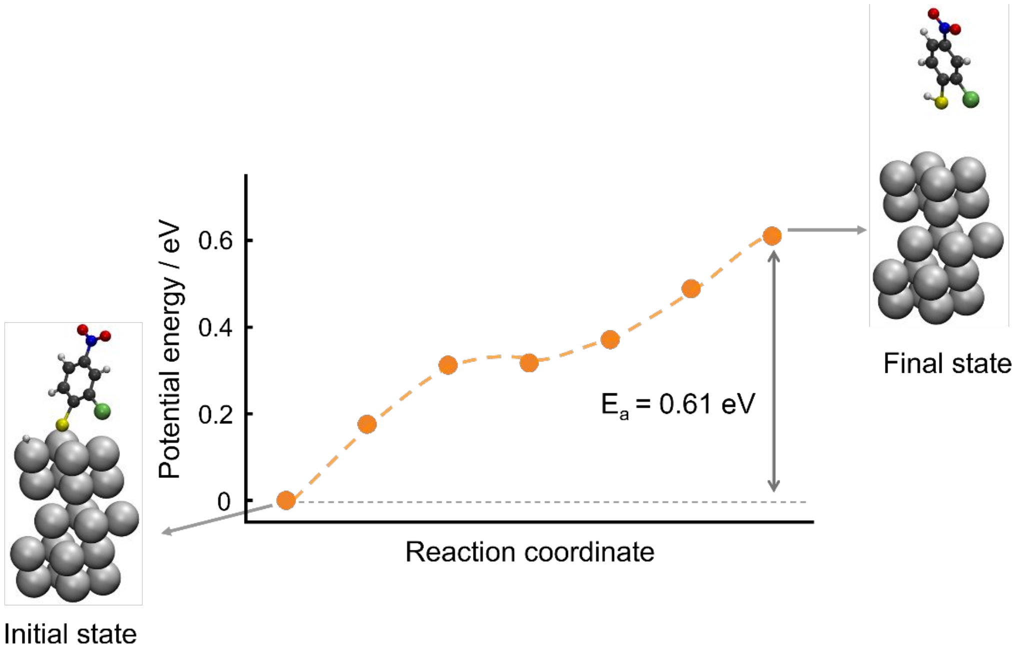

Figure S8. Periodic DFT modeling of CNTP desorption kinetics on Pt(111). Calculated energy profile for the desorption of a CNTP molecule via reaction with a top-site H atom modelled using a 2 × 2 Pt(111) unit cell. The minimum-energy pathway was determined using the CI-NEB method with nine intermediate images. The initial and final states derived from the CI-NEB calculations are shown. The calculated description barrier (E_a) is 0.61 eV, corresponding, via the Arrhenius equation, to a rate constant (k) of 600 s^{-1} and a characteristic desorption timescale (1/k) of 2 ms. Relative to the experimentally observed hydrogenation kinetics, this timescale indicates that CNTP desorption is rapid under ambient conditions and is unlikely to constitute the rate-determining step in the conversion of CNTP to CATP on Pt(111). Atom color scheme: Pt, grey; C, black; H, white; S, yellow; Cl, green; N, blue; O, red.

A GIF illustrating the desorption pathway reconstructed from the nine CI-NEB images is provided as Supporting GIF 1.

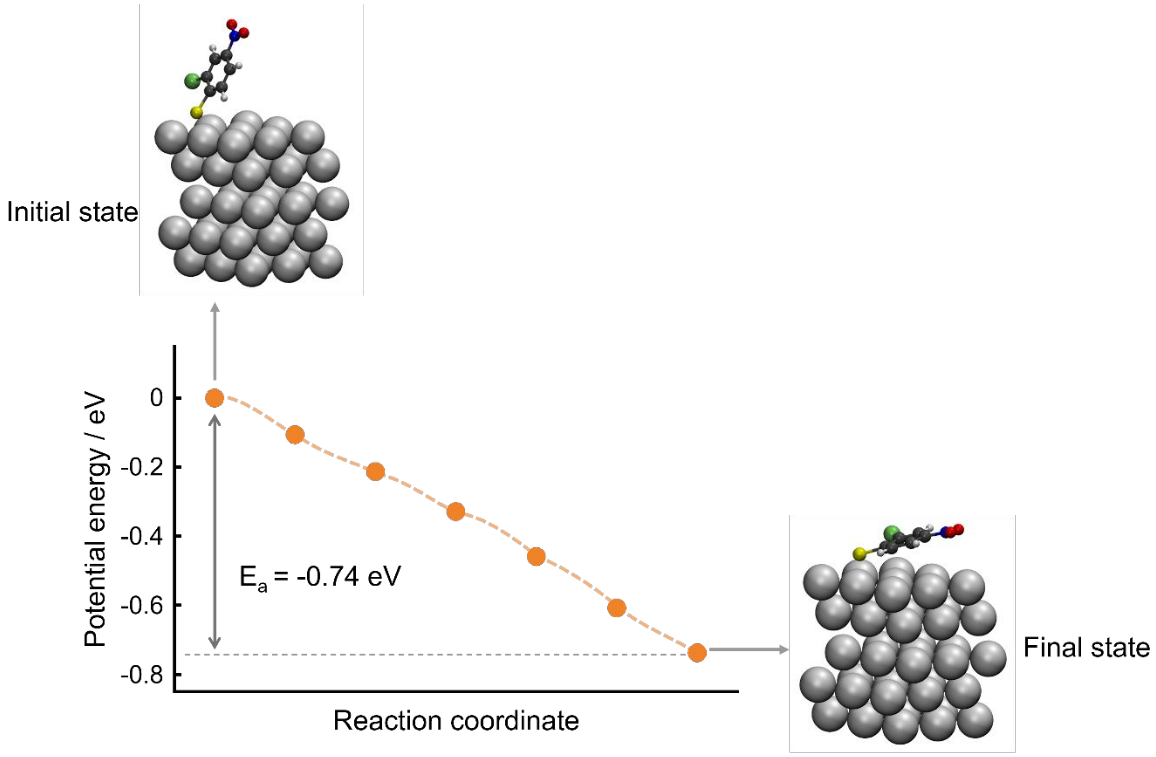

Figure S9. Periodic DFT modeling of CNTP bending kinetics on Pt(111). Calculated energy profile for the bending of a CNTP molecule on a 3 × 3 Pt(111) unit cell, determined using the CI-NEB method with seven intermediate images. The initial and final states derived from the CI-NEB calculations are shown. The calculated bending barrier (E_a) is zero, with an associated enthalpy change (ΔH) of –0.74 eV. These results indicate that CNTP bending occurs spontaneously and is unlikely to represent the rate-determining step in the hydrogenation of CNTP to CATP on Pt(111). Atom color scheme: Pt, grey; C, black; H, white; S, yellow; Cl, green; N, blue; O, red.

A GIF illustrating the bending pathway reconstructed from the CI-NEB images is provided as Supporting GIF 2.

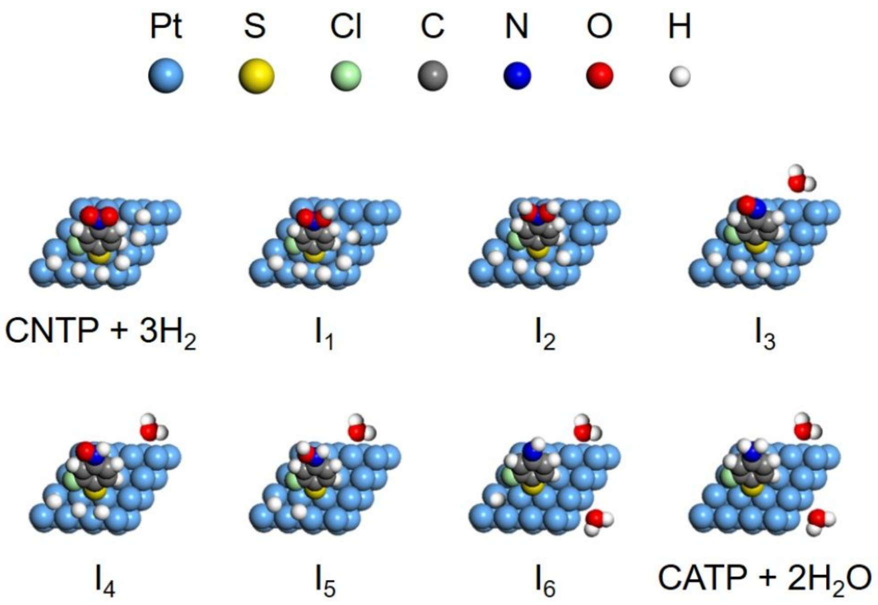

Figure S10. Schematic representations of the hydrogenation steps from CNTP to CATP on Pt(111). Atom color scheme: Pt (cyan), C (grey), S (yellow), Cl (green), N (blue), O (red), H (white).

Supporting GIF files:

Supporting GIF 1: illustrates the CNTP desorption pathway reconstructed from the nine CI-NEB images.

Supporting GIF 2: illustrates the CNTP bending pathway reconstructed from the CI-NEB images.

Supporting GIF 3: illustrates first H-abstraction pathway reconstructed from the CI-NEB images.

Supporting GIF 4: illustrates the second H-abstraction pathway reconstructed from the CI-NEB images.

Supplementary references

1. Clavilier, J.; Faure, R.; Guinet, G.; Durand, R., Preparation of monocrystalline Pt microelectrodes and electrochemical study of the plane surfaces cut in the direction of the {111} and {110} planes. *J. Electroanal. Chem. Interfacial Electrochem.* **1980,** *107* (1), 205-209.
2. Stadler, J.; Schmid, T.; Zenobi, R., Nanoscale Chemical Imaging Using Top-Illumination Tip-Enhanced Raman Spectroscopy. *Nano Lett.* **2010,** *10* (11), 4514-4520.
3. Frisch, M. J.; Trucks, G. W.; Schlegel, H. B.; Scuseria, G. E.; Robb, M. A.; Cheeseman, J. R.; Scalmani, G.; Barone, V.; Petersson, G. A.; Nakatsuji, H.; Li, X.; Caricato, M.; Marenich, A. V.; Bloino, J.; Janesko, B. G.; Gomperts, R.; Mennucci, B.; Hratchian, H. P.; Ortiz, J. V.; Izmaylov, A. F.; Sonnenberg, J. L.; Williams; Ding, F.; Lipparini, F.; Egidi, F.; Goings, J.; Peng, B.; Petrone, A.; Henderson, T.; Ranasinghe, D.; Zakrzewski, V. G.; Gao, J.; Rega, N.; Zheng, G.; Liang, W.; Hada, M.; Ehara, M.; Toyota, K.; Fukuda, R.; Hasegawa, J.; Ishida, M.; Nakajima, T.; Honda, Y.; Kitao, O.; Nakai, H.; Vreven, T.; Throssell, K.; Montgomery Jr., J. A.; Peralta, J. E.; Ogliaro, F.; Bearpark, M. J.; Heyd, J. J.; Brothers, E. N.; Kudin, K. N.; Staroverov, V. N.; Keith, T. A.; Kobayashi, R.; Normand, J.; Raghavachari, K.; Rendell, A. P.; Burant, J. C.; Iyengar, S. S.; Tomasi, J.; Cossi, M.; Millam, J. M.; Klene, M.; Adamo, C.; Cammi, R.; Ochterski, J. W.; Martin, R. L.; Morokuma, K.; Farkas, O.; Foresman, J. B.; Fox, D. J., Gaussian 16 Rev. C.01. *Wallingford CT* **2019**.
4. Hariharan, P. C.; Pople, J. A., The influence of polarization functions on molecular orbital hydrogenation energies. *Theor. Chim. Acta* **1973,** *28* (3), 213-222.
5. Hay, P. J.; Wadt, W. R., Ab initio effective core potentials for molecular calculations. Potentials for K to Au including the outermost core orbitals. *J. Chem. Phys.* **1985,** *82* (1), 299-310.
6. Giannozzi, P.; Baroni, S.; Bonini, N.; Calandra, M.; Car, R.; Cavazzoni, C.; Ceresoli, D.; Chiarotti, G. L.; Cococcioni, M.; Dabo, I.; Dal Corso, A.; de Gironcoli, S.; Fabris, S.; Fratesi, G.; Gebauer, R.; Gerstmann, U.; Gougoussis, C.; Kokalj, A.; Lazzeri, M.; Martin-Samos, L.; Marzari, N.; Mauri, F.;

Mazzarello, R.; Paolini, S.; Pasquarello, A.; Paulatto, L.; Sbraccia, C.; Scandolo, S.; Sclauzero, G.; Seitsonen, A. P.; Smogunov, A.; Umari, P.; Wentzcovitch, R. M., QUANTUM ESPRESSO: a modular and open-source software project for quantum simulations of materials. *J. Phys.: Condens. Matter* **2009,** *21* (39), 395502.

7. Klimeš, J.; Bowler, D. R.; Michaelides, A., Chemical accuracy for the van der Waals density functional. *J. Phys.: Condens. Matter* **2009,** *22* (2), 022201.
8. Berland, K.; Cooper, V. R.; Lee, K.; Schröder, E.; Thonhauser, T.; Hyldgaard, P.; Lundqvist, B. I., van der Waals forces in density functional theory: a review of the vdW-DF method. *Rep. Prog. Phys.* **2015,** *78* (6), 066501.
9. Yin, H.; Zheng, L.-Q.; Fang, W.; Lai, Y.-H.; Porenta, N.; Goubert, G.; Zhang, H.; Su, H.-S.; Ren, B.; Richardson, J. O.; Li, J.-F.; Zenobi, R., Nanometre-scale spectroscopic visualization of catalytic sites during a hydrogenation reaction on a Pd/Au bimetallic catalyst. *Nat. Catal.* **2020,** *3* (10), 834-842.
