# Supplementary figures and images for: Mechanistic Insights into Nitroarene Hydrogenation Dynamics on Pt(111) via In Situ Tip-Enhanced Raman Spectroscopy
Source: J Am Chem Soc. 2025 Oct 17;147(43):39838–45. doi: 10.1021/jacs.5c14338 (PMC12576771; doi:10.1021/jacs.5c14338)

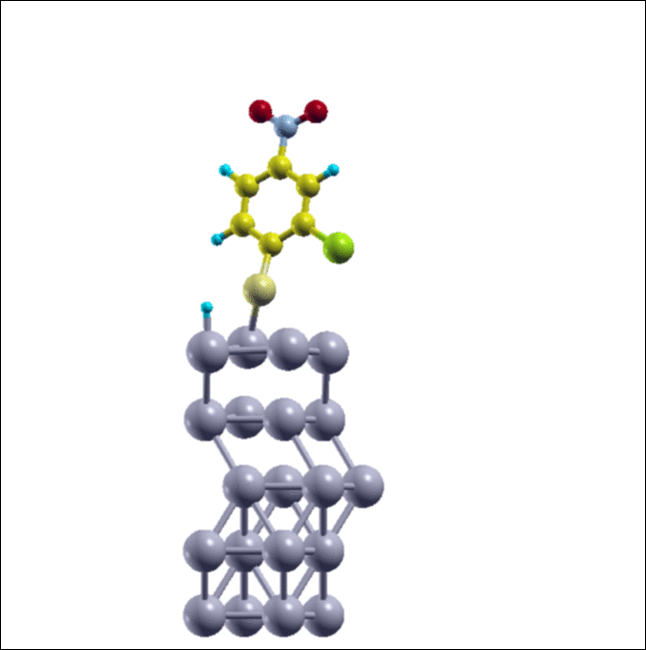

Supplement: Supplementary file 2 [file ja5c14338_si_002.zip › Supporting gif files/Supporting GIF 1.gif]

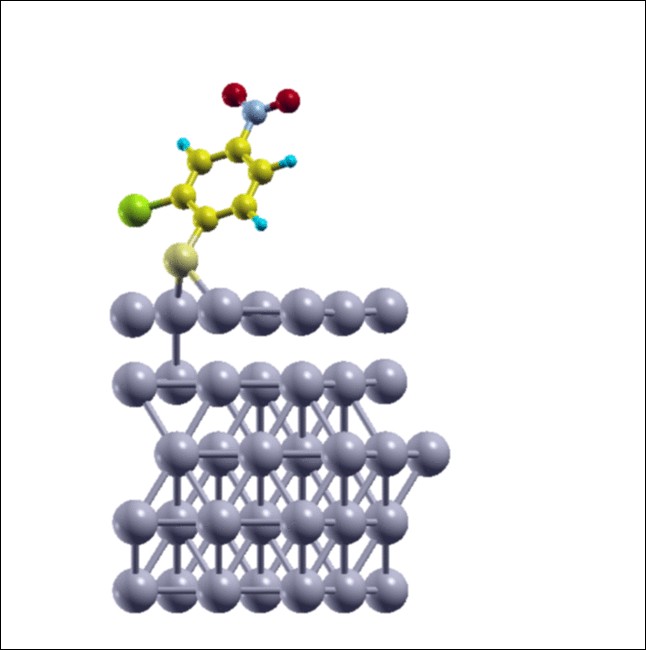

Supplement: Supplementary file 2 [file ja5c14338_si_002.zip › Supporting gif files/Supporting GIF 2.gif]

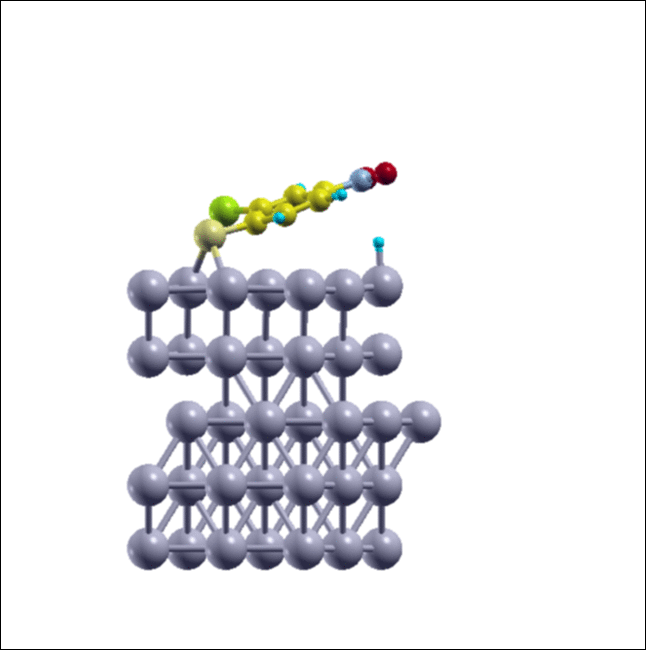

Supplement: Supplementary file 2 [file ja5c14338_si_002.zip › Supporting gif files/Supporting GIF 3.gif]

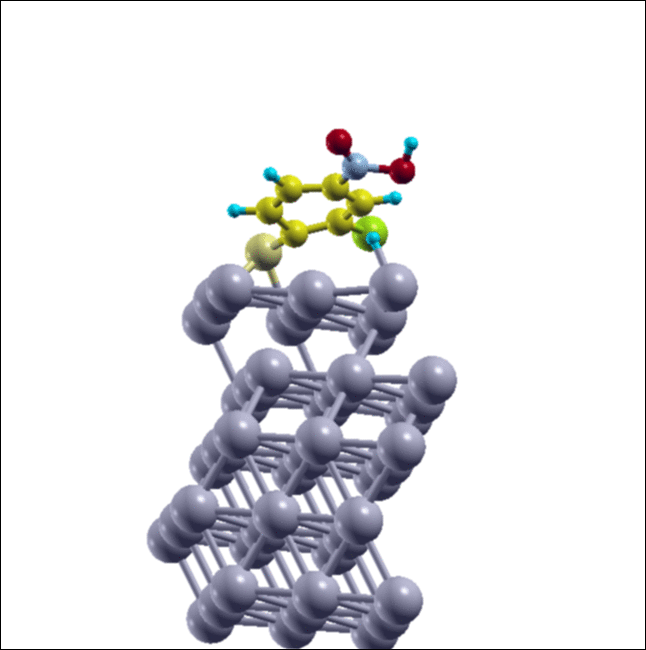

Supplement: Supplementary file 2 [file ja5c14338_si_002.zip › Supporting gif files/Supporting GIF 4.gif]
